# Are high cumulative doses of erythropoietin neuroprotective in preterm infants? A two year follow-up report

**DOI:** 10.1186/s13052-015-0171-1

**Published:** 2015-09-17

**Authors:** R. Luciano, A. Fracchiolla, D. Ricci, F. Cota, V. D’Andrea, F. Gallini, P. Papacci, E. Mercuri, C. Romagnoli

**Affiliations:** Neonatology Unit, Department of Gynecology, Obstetrics and Pediatrics, A. Gemelli University Hospital, Rome, Italy; Pediatric Neurology Unit, Department of Gynecology, Obstetrics and Pediatrics, A. Gemelli University Hospital, Rome, Italy

## Abstract

**Background:**

Preterm infants are at risk for neurodevelopmental sequelae even in absence of major cerebral lesions. The hypothesis that Human Recombinant Erythropoietin (rEpo) could improve the neurodevelopmental outcome in risk neonates has raised the highest interest in recent years.

**Methods:**

A group of preterm neonates born at a gestational age ≤ 30 weeks and free from major cerebral lesions or major visual impairment, were included in the study if they had a complete neurologic evaluation for at least 24 months of postmenstrual age. They were assigned to group I in the case they had been treated with rEpo or group II if untreated. The aim was to evaluate whether rEpo, given at the high cumulative doses utilized for hematologic purposes, is able to improve the neurodevelopmental outcome in preterm infants born at a gestational age ≤ 30 weeks. A group of 104 preterm neonates were studied: 59 neonates who received rEpo for 6.9 ± 2.4 weeks at a median cumulative dose of 6300 UI/Kg (6337 ± 2434 UI/Kg), starting at a median age of 4 days and 45 neonates who were born in the period preceding the routine use of rEpo. The neurodevelopmental quotient at 24 month postmenstrual age was assessed utilizing the Griffiths’ Mental Developmental Scales.

**Results:**

Our results failed to show any difference in the Developmental Quotient at 24 month. Bronchopulmonary dysplasia, minor intraventricular hemorrhages and blood transfusions were the clinical features significantly related to the Developmental Quotient.

**Conclusions:**

Our results do not support the hypothesis that rEpo, administered with the schedule utilized for hematologic purposes, improve the neurodevelopmental outcome of preterm neonates, at least those preterm infants free from major impairments.

## Background

Preterm infants are recognized to be at risk for neurodevelopmental (ND) sequelae even in absence of major cerebral lesions. The abnormal milieu of preterm extrauterine life, in particular ischaemia and inflammation, make preterm infants susceptible to cerebral injury potentially leading to ND sequelae even in absence of major cerebral lesions [1]. Animal studies using a variety of models for hypoxic-ischemic brain injury, as well as a clinical trial on adult humans who had sustained a brain stroke, provided substantial evidence for significant neuroprotective effects of rEpo [2–4]. As a consequence, the hypothesis that rEpo could improve the ND outcome in risk neonates has raised the highest interest in recent years [5–7]. Its neurotrophic, anti-inflammatory, anti-apoptotic, angiogenic, anti-oxidant and anti-epileptic properties have been investigated over the last decade and its underlying mechanisms in terms of signal transduction pathways have been defined [8–18]. Experimental studies have evaluated the neuroprotective effects of high intravenous doses of rEpo given as a fast bolus, but recent retrospective clinical studies have investigated the hypothesis that the high cumulative doses utilized to prevent the anaemia of prematurity might be neuroprotective in preterm infants as well. Conflicting results have been reported. [19–23].

The aim of the present study is to evaluate whether rEpo, given at the high cumulative doses utilized for hematologic purposes, has improved the ND outcome in a series of preterm neonates free from major cerebral lesions or major visual impairment compared with a series of untreated neonates born at similar GA. Secondarily, the clinical variables potentially influencing the outcome have been investigated as well.

## Methods

### Patients

Charts of all preterm infants born at a GA ≤ 30 weeks and admitted to the neonatal intensive care unit of the A. Gemelli University Hospital in Rome from 12.1.2002 to 1.31.2007 were retrospectively evaluated. Infants with congenital anomalies or major neurological and sensory impairments (cerebral palsy, deafness, retinopathy of prematurity stage >2) as well as infants affected by major intracranial lesions, i.e. extensive subependymal-intraventricular hemorrhage (SEH-IVH Grade III-IV) [24], periventricular infarction, periventricular leucomalacia and post-hemorrhagic hydrocephalus or severe ventricular dilation, were excluded from the study. The occurrence of minor SEH-IVH (Grade I-II) [24] was not considered among the exclusion criteria. Serial ultrasound evaluations (at least three times during the first week of life and weekly thereafter until discharge) were performed using a Hewlett-Packard Image Point equipped with a multifrequency beam [5–7,5 MHz). Diagnosis of bronchopulmonary dysplasia (BPD) included O2 dependency at 36 week postmenstrual age (PMA) and / or more than 28 days of oxygen therapy [25]. No major changes in clinical care were introduced during the study period. Two groups of neonates were considered: the study group (G1) includes neonates born between December 2004 and January 2007 who received rEpo for preventing the anaemia of prematurity; the control group (G2) includes neonates born during the 2 years before the protocol for prevention of anaemia was started. Approval for the study was obtained by the Ethical Commettee of Gemelli University Hospital.

### rEpo therapy

A dose of 300 UI/kg 3 times per week was administered subcutaneously or intravenously in 24 h until central venous line was removed and then subcutaneously [26]. The rEpo was started before the 14^th^ day of life and continued through the 35^th^ week PMA [27] unless adverse effects were observed.

### Follow-up

All infants have been regularly followed for at least 24 months PMA with a standardized neurological and ND assessment. Neurological examination consisted of a structured assessment evaluating cranial nerve function, posture, movements, tone, reflexes/saving reactions and visual behaviour [28].

ND assessment at 24 month PMA was performed utilizing the Griffiths’ Mental Developmental Scales [29]. The assessment includes motor, personal and social, language, hand and eye and performance scales. ND was classified as: normal, when developmental quotient (DQ) was > 85; borderline, when DQ was between 70 and 85; delayed when DQ was less than 70. Neurological examination as well as ND assessment was always performed by trained pediatric neurologists.

### Statistical analyses

The data are reported as mean ± standard deviation or count and percent according to variables nature (continuous or count/categorical). The study population has been divided in two groups according to the rEpo administration. The differences between groups were evaluated by the Wilcoxon rank sum test (Mann Whitney *U* test) or the Student's *t*-test as appropriate according to nonparametric/parametric distribution for the continuous variables; categorical data were analyzed by Fisher’s exact test.

The association between the studied variables and the DQ has been investigated by Pearson’s correlation for continuous variables (r for significant associated variables have been reported) or the Wilcoxon rank sum test/student’s *t*-test for the binary ones. Linear regression has been used to adjust the significant associations found for birth weight (BW) or GA; GA and BW were collinear, so we cannot put both in the same model. A high grade of collinearity was found for mechanical ventilation, oxygen and BPD; we decided to use only the last one in the multivariate analysis. When 2 variables described the same risk (e.g., transfusion number and transfusion requirement), the variables that had the stronger association with the ND outcome were chosen for multivariate analysis. A two tailed p < 0.05 was considered significant. Analyses were performed with Stata/IC 12.

## Results

One hundred and four infants were included in the study. They represented the 87 % of the infant series from the original data set with complete medical records and follow-up evaluation once the exclusion criteria were applied. G1 includes 59 and G2 45 neonates. The main clinical variables of both groups are described in Table 1. We found a higher prevalence of surfactant treatment in G1 and a higher percentage of minor SEH-IVH in G2. Treated infants received rEpo at a median cumulative dose of 6300 UI/Kg (6337 ± 2434 UI/Kg), starting at a median age of 4 days (range: 1–13 days). The median duration of treatment was 6.9 ± 2.4 weeks. A dose of 300 UI/kg 3 times per week was administered subcutaneously or intravenously in 24 h. Thirty-five neonates received rEpo intravenously until central venous line was removed and then subcutaneously, 4 neonates received rEpo intravenously till the treatment was discontinued. rEpo was administered subcutaneously from the start in 20 neonates. The ND assessment was performed at 24 ± 1 month PMA. The median total DQ score was 101.0 ± 11.7 for G1 and 108.9 ± 13.0 for G2 (*p* = 0.538) (Table 2). There is no difference in the prevalence of total DQ ≤ 85 at 24 month PMA in the two groups (Table 3). We only found a higher incidence of DQ ≤ 85 at 24 month PMA in the A Griffiths’ scale in G2 (*p* = 0.013). Two neonates in G1 had DQ < 70.

**Table 1:** Main clinical variables in the two studied groups

		G1	G2	*p*
		*N* = 59	*N* = 45	
Female	(*n* - %)	40 – 67.8	22 – 48.9	0.07
Twins	(*n* - %)	12 – 20.3	15 – 33.3	0.176
Gestational age	(M ± SD) wk	28.1 ± 1.7	28.3 ± 1.2	0.591
Birth weight	(M ± SD) g	1110.8 ± 304.6	1099.8 ± 229.0	0.840
Vaginal delivery	(*n* - %)	7 – 11.9	3 – 6.7	0.508
5’ Apgar score >7	(*n* - %)	56 – 94.9	42 -93.3	1.000
Prenatal steroid treatment (*n* - %)		31 – 52.5	26 – 57.8	0.692
Postnatal steroid treatment				
* n* - %		8 – 13.6	6 – 13.3	1.000
M ± SD, d		0.9 ± 2.4	1.9 ± 8.2	0.361
Surfactant treatment (*n* - %)		56 – 94.9	28 – 62.2	<0.01
CPAP (M ± SD), h		319.4 ± 311.4	274.4 ± 221.4	0.412
Conventional mechanical ventilation, (M ± SD), h		80.7 ± 121.2	60.0 ± 104.4	0.361
HFOV (M ± SD), h		54.8 ± 139.8	22.8 ± 92.7	0.187
O2 therapy (M ± SD), h		281.5 ± 395.1	330.0 ± 443.1	0.558
Inhaled nitric oxide(M ± SD), h		2.8 ± 15.5	3.9 ± 15.5	0.708
Bronchopulmonary dysplasia (*n* - %)		32 – 54.2	18 – 40.0	0.169
Indomethacin treatment (*n* - %)		7 – 11.9	1 – 2.2	0.133
Patent ductus arteriosus ligation (*n* - %)		4 – 6.8	0 – 0.00	0.131
Packed red blood cells transfusions				
* n* - %		36 – 61.0	31 – 68.9	0.535
M ± SD		1.4 ± 1.5	1.4 ± 1.5	0.850
Retinopathy of prematurity grade I-II (*n* - %)		37 – 62.7	20 – 44.4	0.075
Intracranial hemorrhage grade I-II (*n* - %)		22 – 37.3	28 – 62.2	0.017
Intracranial hemorrhage grade I (*n* - %)		6 – 10.2	6 – 13.3	0.759
Intracranial hemorrhage grade II (*n* - %)		16 – 27.1	22 – 48.9	0.026
Necrotizing enterocolitis (*n* - %)		1 – 1.7	0 – 0.0	1.000

*h* hours

**Table 2:** Developmental quotient (DQ) according to the Griffith scales in the two studied groups

	G1	G2	*p*
	(M ± SD)	(M ± SD)	
A – loco-motor	105.4 ± 13.9	103.4 ± 13.1	0.453
B – personal-social	112.4 ± 14.7	111.6 ± 18.4	0.816
C – hearing and language	103.1 ± 18.7	96.8 ± 15.3	0.069
D – eye and hand coordination	102.0 ± 12.3	102.6 ± 13.9	0.823
E – performance	105.9 ± 17.1	108.3 ± 16.5	0.480
DQ total	101.0 ± 11.7	108.9 ± 13.0	0.538

**Table 3:** Number of neonates with developmental quotient (DQ) ≤85 in the two studied groups

	G1	G2	*p*
	(M ± SD)	(M ± SD)	
A – loco-motor	0 – 0	5 – 11.1	0.013
B – personal-social	1 – 1.7	3 – 6.7	0.313
C – language	9 – 15.3	6 – 13.3	1.000
D – eye and hand coordination	4 – 6.8	3 – 6.7	1.000
E – performance	6 – 10.2	4 – 8.9	1.000
Total DQ	3 – 5.1	3 – 6.7	1.000

Clinical variables showing significant correlation with the DQ were number of blood transfusions, lower BW and GA, hours of oxygen therapy and conventional mechanical ventilation (Table 4). When DQ was evaluated according to the presence/absence of clinical variables, no significant difference was found according to surfactant therapy (DQ respectively 103.9 ± 12.1 and 109.8 ± 14.7 in treated and not treated infants p 0.071). On the contrary, BPD, minor SEH-IVH and requirement of blood transfusion were significantly associated with lower DQ scores (Table 5). The correlation was still present after adjustment for BW in the case of BPD and minor SEH-IVH and after adjustment for GA as concerns BPD. A subgroup analysis for those subgroups showing statistically significant differences in DQ was performed. No significant difference in DQ according to exposure or not to rEpo was found (Table 6).

**Table 4:** Clinical variables showing significant correlation with the Developmental Quotient (DQ) (Pearson’s correlation for continuous variables)

	*r*	*p*
Gestational age	0.2175	<0.05
Birth weight	0.2443	<0.05
Conventional mechanical ventilation, h	- 0.2805	<0.01
O2 therapy, h	- 0.2130	<0.05
Packed red blood cells transfusions, n	- 0.2890	<0.01

*h* hours

**Table 5:** Developmental Quotient (DQ) according to the presence/absence of clinical variables (only clinical variables showing statistically significant differences are reported)

	*Present*	*Absent*	*p*
	(M ± SD)	(M ± SD)	
Bronchopulmonary dysplasia	102.9 ± 12.6	109.2 ± 13.3	0.019
Packed red blood cells transfusions	101.7 ± 11.7	108.9 ± 13.0	0.018
Subependimal-intraventricular hemorrhage grade I-II	101.8 ± 13.4	108.2 ± 12.3	0.011

**Table 6:** Subgroup analysis showing Developmental Quotient (DQ) according to exposure or not to rEpo (only those subgroups showing statistically significant differences are reported)

	*rEpo+*	*rEpo-*	*p*
	(M ± SD)	(M ± SD)	
Bronchopulmonary dysplasia	102.0 ± 9.8	99.2 ± 14.65	0.411
Packed red blood cells transfusions	104.3 ± 9.7	101.2 ± 15.2	0.312
Subependimal-intraventricular hemorrhage grade I-II	103.4 ± 11.5	100.5 ± 14.7	0.454

## Discussion

The brain of preterm infants is highly susceptible to ischemic and inflammatory insults that may be responsible for ND sequelae even in absence of major cerebral lesions. Erythropoietin is considered a promising neuroprotective compound for its known antiinflammatory and antiapoptotic properties. It is also routinely used in Neonatology Units for haemathologic purposes. EPO has been administered in 2 different ways. A previous study from our group was performed to compare the two ways of administration. No difference was found in the hematologic effect [26]. Our results failed to show any difference in DQ at 24 month PMA between preterm neonates treated or not treated with rEpo given at doses commonly administered for preventing the anaemia of prematurity, whereas we found a significant lower DQ in neonates affected by minor IVH or BPD and in neonates who required blood transfusions. Similar results on the neuroprotective effect of the rEpo treatment utilized for hematologic purposes were found by the first authors who tested this hypothesis [19, 20]. Other studies reported a better cognitive outcome following a higher rEpo dosage in preterm neonates compared with a lower dosage group [21, 22]. Bierer et al., who analyzed a small group of 12 preterm infants, observed higher mental developmental index (MDI) scores in those neonates with serum erythropoietin concentrations ≥ 500 mU/mL compared with neonates whose serum concentrations was < 500 mU/mL [21]. Psycomotor developmental index scores did not differ between those 2 groups. Following this study, Brown et al. examined the association between different doses of rEpo treatment and ND outcomes in a large number of preterm infants ≤ 30 week GA [22]. They found that higher 6-week cumulative rEpo doses were associated with higher MDI scores, consistent with the results reported by Bierer. An important limitation of this study was that the final population was only 22 % of the original cohort. However, the wide range of cumulative rEpo doses included in the statistical analysis helped to establish a dose–response relationship between rEpo doses and ND outcomes (MDI scores), and it allowed for the hypothesis of a threshold effect. The rEpo per Kg doses in our population were intermediate between the two groups in Brown’s study, but the treated babies in our series received a larger cumulative dose than the higher dosage group in Brown’s study because of a longer duration of treatment. In 2010, Neubauer et al. investigated the ND outcome at school age of extremely low birth weight (ELBW) infants submitted to rEpo treatment in order to stimulate erythropoiesis [23]. Analyses of variance (ANOVAs) showed that rEpo group scored significantly better than untreated children. Similar results were obtained when those children with SEH-IVH were considered, whereas treated and untreated children without SEH-IVH did not differ in their outcome. As a consequence, differences found in the general population should be credited to children with SEH-IVH. The results of this observational study support the hypothesis that rEpo is neuroprotective in ELBW infants with SEH-IVH, but not effective as well in preterm neonates without cerebral lesions. These findings could contribute to explain some conflicting results reported in the different studies and are consistent with our results obtained in preterm infants not affected by intracranial lesions. We found a significant lower DQ in neonates affected by minor IVH or BPD and in neonates who required blood transfusions. The present study failed to show a neuroprotective effect of high cumulative doses of rEpo in preterm infants without major cerebral lesions, at least for a ND assessment limited to 24 month PMA. The number of infants considered for the study is limited because criteria for exclusion were strict in order to eliminate potential bias. Moreover, neonates were not included if they had not been subjected to a complete neurological assessment with developmental scales at the correct time. We are conscious that number of patients is not sufficient to draw definitive conclusions but our results could be helpful when regarded together with similar analysis in the literature.

Since studies dealing with this topic, including the present one, were not originally designed to examine ND outcomes, the results should be regarded as preliminary information for better targeting future investigations.

## Conclusions

The present study failed to document a neuroprotective effect of high cumulative doses of rEpo in PT infants without major cerebral lesions, at least for a ND assessment limited to 24 month CA. Although the limited number of patients included in our study do not allow to draw definitive conclusions, our results may be helpful when regarded together with similar analysis in the literature.
